# Metabolic Landscape of Endometrial Cancer: Insights into Pathway Dysregulation and Metabolic Features

**DOI:** 10.3390/biomedicines14010202

**Published:** 2026-01-17

**Authors:** Qing Yang, Xiaoli Tian, Min Hu, Wenjing Ma, Qingzhen Xie, Jingchun Liu, Li Hong

**Affiliations:** 1 Department of Gynecology and Obstetrics, Renmin Hospital of Wuhan University, Wuhan 430060, China; 2 Department of Pathology, Renmin Hospital of Wuhan University, Wuhan 430060, China; 3 Centre for Reproductive Medicine, Renmin Hospital of Wuhan University, Wuhan 430060, China

**Keywords:** endometrial cancer, metabolism, machine learning

## Abstract

**Background:** Metabolic reprogramming is increasingly recognized as a hallmark of endometrial cancer, yet tissue-based metabolic signatures remain insufficiently defined. **Methods:** Untargeted metabolomics was performed on paired endometrial cancer (*n* = 10) and adjacent normal tissues (*n* = 10). Differential metabolites were identified through multivariate and univariate analyses. KEGG enrichment characterized altered pathways, while Random Forest and SVM were used for machine-learning-based feature prioritization. ROC analyses were conducted to evaluate the discriminative potential of selected metabolites. **Results:** 300 metabolites were significantly altered. Tumor tissues showed increased sphingolipid metabolism, glutathione metabolism, and arachidonic acid metabolism, alongside decreased bile acid, phenylalanine, and steroid biosynthesis. Machine learning converged on six key metabolites that demonstrate strong tissue-discriminative capacity. **Conclusions:** Endometrial cancer exhibits a distinct metabolic profile characterized by lipid remodeling and redox adaptation. The six metabolites identified through machine-learning-based analyses represent candidate metabolic features associated with endometrial cancer and provide a foundation for future mechanistic studies and validation in larger, independent cohorts.

## 1. Introduction

Endometrial cancer represents a malignancy whose incidence continues to rise disproportionately to other gynecologic tumors, reflecting not only demographic shifts but also deeper biological vulnerabilities within the endometrial microenvironment [1,2,3]. Although genetic and hormonal drivers of this disease have been extensively explored, accumulating evidence suggests that malignant transformation in the endometrium cannot be fully understood without examining the metabolic state that accompanies and enables these molecular alterations. Growing evidence shows that metabolic alterations can reshape redox balance, modify membrane lipid composition, influence nucleotide biosynthesis, and regulate oncogenic signaling pathways [4,5,6]. These changes ultimately affect tumor initiation, progression, immune evasion, and therapeutic response [7,8,9]. Understanding these metabolic alterations has therefore become an essential component of deciphering the biological drivers of endometrial cancer.

While several studies have reported serum-based metabolic alterations in endometrial cancer, body-fluids-derived profiles do not fully recapitulate the metabolic ecosystem of tumor tissues [10]. Moreover, published research has predominantly focused on specific metabolic pathways, lacking a systematic analysis of the entire metabolic network [11,12,13,14]. Consequently, the architecture of metabolic reprogramming within primary tumors, the coordination among distinct pathways, and the identity of key metabolic nodes with disease-associated or mechanistic significance remain poorly defined.

Advances in untargeted metabolomics offer an opportunity to address these gaps by enabling simultaneous detection of hundreds of metabolites directly within tumor tissues. Yet, interpreting such high-dimensional data remains challenging. Integrating metabolomic profiling with machine-learning algorithms provides a powerful strategy to identify reproducible and biologically meaningful metabolic features, thereby improving the interpretation of metabolic circuitry and prioritization of candidate features for further investigation. Nevertheless, metabolomics-based discovery is also subject to inherent limitations, including analytical variability, challenges in biological interpretation, and the risk of overinterpretation in the absence of independent validation, which underscores the importance of cautious interpretation and validation-oriented study design [15,16,17].

In this study, we performed a comprehensive untargeted metabolomic analysis of paired endometrial cancer and adjacent normal tissues. By integrating differential metabolite profiling, pathway enrichment, and two independent machine-learning approaches, we aimed to characterize the metabolic architecture associated with malignant endometrium. Our findings delineate a distinct tissue-level metabolic landscape involving sphingolipid, glutathione, and arachidonic acid metabolism. These results refine our understanding of metabolic reprogramming in endometrial cancer and identify a set of metabolites with consistent tissue-discriminative capacity, providing a foundation for future mechanistic studies and validation-oriented translational research.

## 2. Materials and Methods

### 2.1. Clinical Samples

From September 2024 to September 2025, a total of 10 pairs of tissue specimens were collected at Renmin Hospital of Wuhan University (Wuhan, China), including 10 endometrial cancer tissues and 10 matched adjacent normal endometrial tissues from surgery. All participants had no history of metabolic disorders, cardiovascular disease, hypertension, and none had received neoadjuvant chemotherapy or radiotherapy prior to surgery. Postoperative pathological examination confirmed all tumor specimens as endometrioid adenocarcinoma. Immediately after excision, all tissues were snap-frozen and stored at −80 °C until analysis. This study was approved by the Ethics Committee of Renmin Hospital of Wuhan University (No. WDRY2023-K137), and written informed consent was obtained from all patients.

### 2.2. Metabolite Extraction and Metabolite Identification

In brief, approximately 30 mg of tissue was homogenized, and metabolites were extracted using a methanol–acetonitrile mixture (1:1, *v*/*v*). Untargeted metabolomic profiling was performed on a Thermo Orbitrap Exploris 120 mass spectrometer. Metabolite identification was carried out using the PSNGM Database (Paisenon, Shanghai, China), which integrates multiple libraries, including an in-house standard library, mzCloud (https://www.mzcloud.org/, accessed on 15 November 2025), LIPID MAPS (https://www.lipidmaps.org/, accessed on 15 November 2025), HMDB (https://hmdb.ca/, accessed on 15 November 2025), MoNA (https://mona.fiehnlab.ucdavis.edu/, accessed on 15 November 2025), NIST 2020 MS/MS, and AI-predicted MS/MS spectral libraries. Raw data were converted using ProteoWizard (version 3.0.8789), and peak deconvolution, filtering, and alignment were performed using XCMS (version 3.12.0).

### 2.3. Quality Control (QC), Principal Component Analysis (PCA), Partial Least Squares-Discriminant Analysis (PLS-DA), and Orthogonal Partial Least Squares-Discriminant Analysis (OPLS-DA)

QC samples were prepared by pooling equal aliquots from all study samples and were used to monitor instrument stability and data reliability throughout the run. One QC sample was injected after every five study samples. Consistency among QC injections was evaluated using Pearson correlation analysis. PCA was performed to visualize the clustering of QC and study samples, and plots were generated using the ggplot2 package (version 3.4.4). PLS-DA and OPLS-DA analyses were performed according to sample group assignments. PLS-DA distinguishes groups by decomposing the X and Y matrices, whereas OPLS-DA further removes orthogonal components unrelated to class separation, thereby enhancing interpretability by concentrating group-related variance into the first predictive component. Model performance was assessed using R2X, R2Y, and Q2 values, where values approaching 1 indicate stronger goodness-of-fit and predictive capability.

### 2.4. Differential Metabolites Analysis and Pathway Enrichment Analysis

Differential analysis was performed on all detected metabolites using the criteria VIP >1, *p* < 0.05, and fold change ≥2 or ≤0.5. The resulting differential metabolites were subsequently subjected to KEGG pathway enrichment analysis. Enrichment significance was evaluated based on the Rich factor, *p* value, and the number of differential metabolites mapped to each pathway. The Rich factor was defined as the ratio of differential metabolites enriched in a pathway to the total number of annotated metabolites within that pathway, with higher values indicating stronger enrichment.

### 2.5. Random Forest Analysis, Support Vector Machine (SVM) and Receiver Operating Characteristic (ROC) Curve Analysis

Machine-learning approaches were applied for exploratory feature selection and prioritization. Machine-learning analyses, including Random Forest and SVM models, were performed using the mlr3verse package (version 0.2.7). Metabolites that were consistently identified as important features by both algorithms were subjected to ROC curve analysis using the pROC package (version 1.18.2). The area under the curve (AUC) was calculated to evaluate discriminatory performance.

### 2.6. Statistical Analysis and Software

Student’s *t*-test was used to compare differences between the two groups. Correlation analyses were performed using the Pearson method. Statistical visualizations were generated using GraphPad Prism (version 10.1.2, GraphPad Software, San Diego, CA, USA) or R software (version 4.0.3, R Foundation for Statistical Computing, Vienna, Austria). *p* < 0.05 was considered statistically significant.

## 3. Results

### 3.1. Clinical Characteristics of the Patients

A total of ten patients with endometrial cancer who underwent surgical resection were enrolled in this study. For each patient, paired samples of tumor tissue and the corresponding adjacent normal tissue were collected for metabolomic analysis. The mean age of the patients was 54.2 ± 9.2 years. According to the FIGO classification, 5 patients were diagnosed with stage I, 2 with stage II, and 3 with stage III. Histologically, all tumors were confirmed as endometrioid adenocarcinoma. No patients received neoadjuvant chemotherapy or radiotherapy prior to sample collection. The detailed clinical characteristics of the patients are summarized in Supplementary Table S1.

### 3.2. Quality Control Assessment of the Metabolomics Data

**Figure 1:**
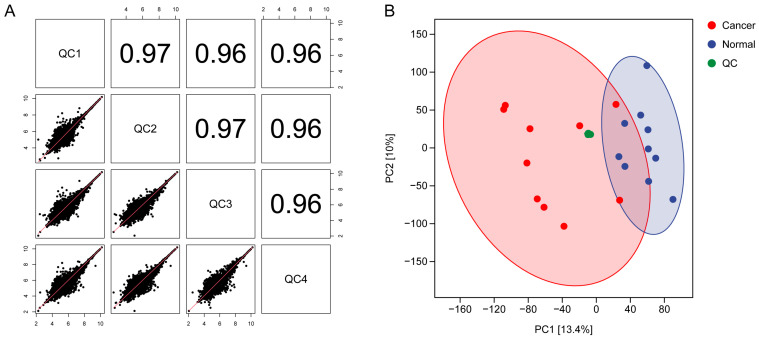
QC assessment. (**A**) Pearson correlation heatmap of QC samples demonstrating high inter-QC consistency. (**B**) PCA score plot showing tight clustering of QC samples and clear separation between tumor and adjacent tissues.

To evaluate the stability of the LC-MS system and the reproducibility of the metabolomic measurements, four pooled QC samples were injected throughout the analytical sequence. Pairwise correlation analysis demonstrated extremely high Pearson correlation coefficients among the QC injections (range: 0.96–0.97), indicating excellent signal consistency and analytical reproducibility (Figure 1A). PCA further supported the robustness of the dataset. The QC samples clustered tightly together, confirming minimal batch effect and high analytical stability. In addition, a clear separation trend was observed between endometrial cancer tissues and adjacent non-tumor tissues along the major principal components, suggesting intrinsic metabolic differences between the two groups (Figure 1B). These results indicate that the dataset is of high quality and appropriate for subsequent supervised multivariate analyses.

### 3.3. Supervised Multivariate Analyses Reveal Distinct Metabolic Profiles Between Tumor and Adjacent Tissuess

**Figure 2:**
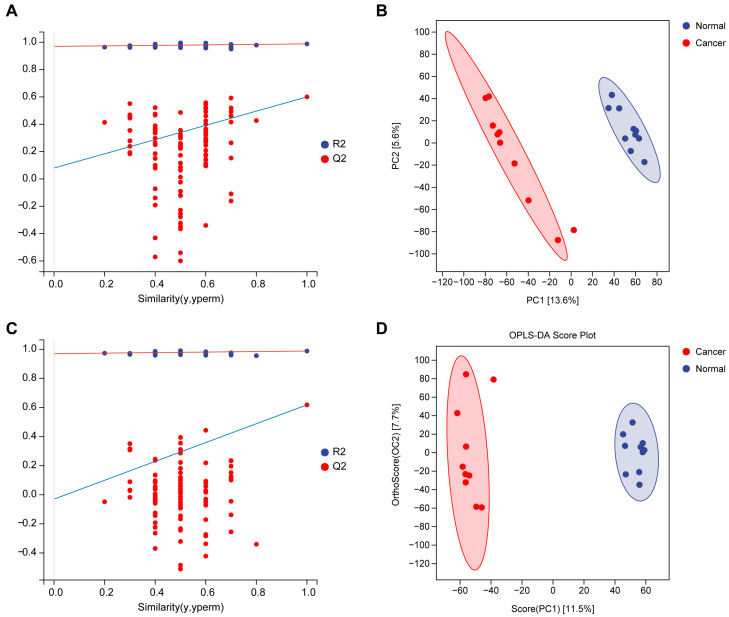
PLS-DA and OPLS-DA multivariate statistical analyses. (**A**) Permutation test of the PLS-DA model demonstrating no overfitting and good model reliability. (**B**) PLS-DA score plot showing clear discrimination between tumor and adjacent tissues. (**C**) Permutation test of the OPLS-DA model confirming robustness and predictive capability. (**D**) OPLS-DA score plot with distinct separation of the two groups.

To further investigate metabolic differences between endometrial cancer tissues and adjacent non-tumor tissues, supervised multivariate analyses were performed. PLS-DA demonstrated a clear separation between the two groups (Figure 2A,B), indicating substantial metabolic reprogramming associated with malignant transformation. The PLS-DA model showed reliable discriminative performance, with R^2^X = 0.192, R^2^Y = 0.988, and Q^2^ = 0.600. OPLS-DA was subsequently conducted to remove variations unrelated to class separation. OPLS-DA further enhanced the distinction between tumor and adjacent tissues with R^2^X = 0.192, R2Y = 0.988, and Q^2^ = 0.618 (Figure 2C,D), confirming that cancer-related metabolic alterations dominate the dataset. These supervised analyses together demonstrate that endometrial cancer samples possess a metabolic phenotype clearly distinct from that of adjacent normal tissues.

### 3.4. Metabolite Identification, Chemical Classification, and Differential Metabolite Screening

**Figure 3:**
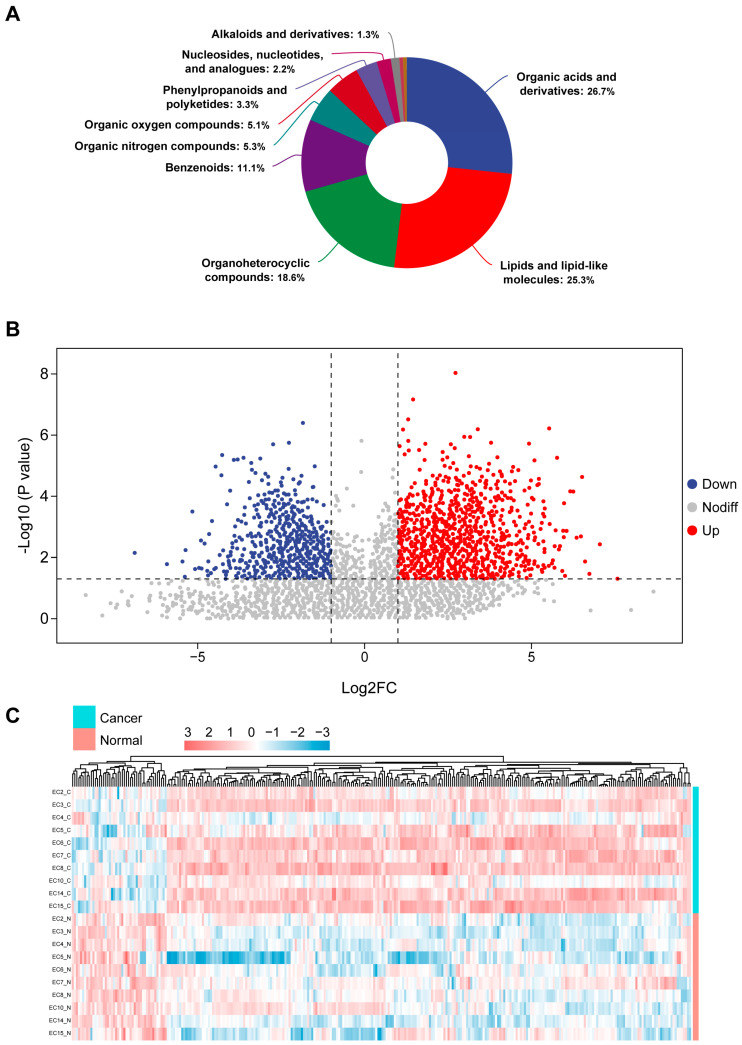
Identification and visualization of differential metabolites. (**A**) Composition distribution of detected metabolites categorized by chemical class. (**B**) Volcano plot illustrating statistically significant differential metabolites, with upregulated metabolites in red and downregulated metabolites in blue. (**C**) Heatmap showing expression patterns of differential metabolites across the paired samples.

A total of 871 metabolites were annotated through accurate mass matching, MS/MS fragmentation pattern comparison, and database searching, with high-confidence metabolites assigned MSI Level 2 identification. Chemical classification revealed that organic acids and derivatives and lipid-related metabolites constituted the largest categories (Figure 3A). Differential metabolites between tumor and adjacent tissues were identified using combined multivariate and univariate criteria (VIP > 1, *p* < 0.05, fold change ≥2 or ≤0.5). A total of 300 significantly altered metabolites were obtained. The volcano plot highlights the distribution of upregulated and downregulated metabolites (Figure 3B). Heatmap visualization of differential metabolites further demonstrated distinct expression patterns between the two groups (Figure 3C). These results indicate pronounced metabolic reprogramming in endometrial cancer.

### 3.5. Pathway Enrichment Analysis of Differential Metabolites

**Figure 4:**
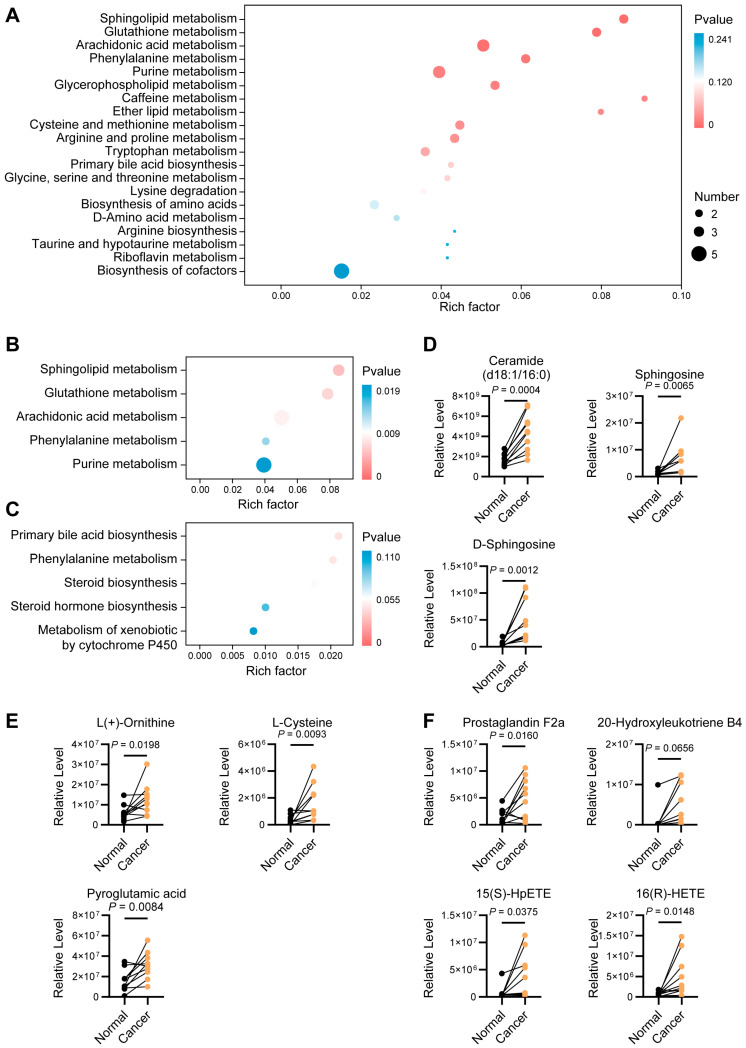
KEGG pathway enrichment analysis. (**A**) KEGG enrichment of all differential metabolites. (**B**) Enriched pathways associated with upregulated metabolites. (**C**) Enriched pathways associated with downregulated metabolites. (**D**) Significantly upregulated metabolites within the sphingolipid metabolism pathway. (**E**) Significantly upregulated metabolites within the glutathione metabolism pathway. (**F**) Significantly upregulated metabolites within the arachidonic acid metabolism pathway.

Pathway enrichment analysis integrating all differential metabolites, together with their up-regulated and down-regulated subsets, was performed to characterize the metabolic network alterations in endometrial cancer. In the overall enrichment profile (Figure 4A), significantly enriched pathways encompassed lipid, amino acid, carbohydrate, and redox-related metabolism, indicating a multilayered and multidirectional metabolic reprogramming in tumor tissues. When metabolites were analyzed separately according to their direction of change, more specific biological patterns emerged. Up-regulated metabolites were predominantly enriched in sphingolipid metabolism, glutathione metabolism, and arachidonic acid metabolism, reflecting an enhanced reliance on polyunsaturated lipid-derived signaling and redox buffering systems within tumor tissues (Figure 4B). In contrast, metabolites involved in primary bile acid biosynthesis, phenylalanine metabolism, and steroid biosynthesis were decreased, suggesting suppression of bile acid-related cholesterol catabolism, reduced aromatic amino-acid turnover, and attenuated steroidogenic flux (Figure 4C).

Further examination of metabolites within the top three up-regulated pathways provided more detailed insights. In the sphingolipid pathway, ceramide, sphingosine, and D-sphingosine were markedly increased (Figure 4D). In the glutathione pathway, ornithine, L-cysteine, and pyroglutamic acid were elevated in tumor tissues (Figure 4E). Within arachidonic acid metabolism, the up-regulated metabolites prostaglandin F2α, 20-hydroxyleukotriene B4, 15(S)-HpETE, and 16(R)-HETE indicate enhanced production of eicosanoids and hydroxyeicosatetraenoic acids, consistent with a more pro-inflammatory and protumorigenic lipid mediator milieu in endometrial cancer (Figure 4F).

### 3.6. Machine-Learning-Based Feature Selection Identifies Robust Metabolic Features

**Figure 5:**
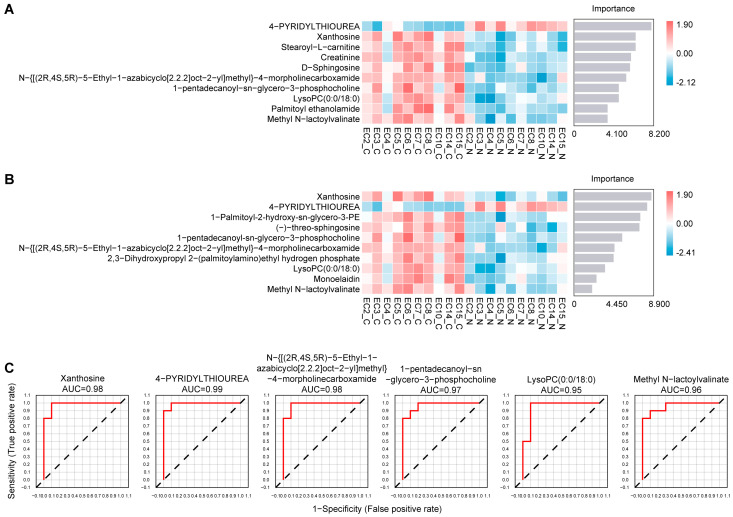
Machine-learning-based metabolite selection and ROC analysis. (**A**) Top 10 important metabolites identified by the Random Forest model. (**B**) Top 10 metabolites identified by the SVM model. (**C**) ROC curves of the six metabolites jointly selected by both algorithms, with AUC values illustrating their tissue-discriminative capacity.

To refine the identification of metabolites most strongly associated with endometrial cancer, we employed two complementary machine-learning algorithms, Random Forest and SVM, each of which provides distinct mathematical advantages in high-dimensional metabolomics datasets. The RF model, leveraging ensemble decision-tree voting, ranked metabolites according to mean decrease in accuracy, while the SVM model identified features with the greatest classification weights within the optimized hyperplane (Figure 5A,B). Despite the methodological differences between these two algorithms, both consistently highlighted a subset of metabolites with high discriminatory power. Notably, six metabolites were shared among the top 10 features identified independently by RF and SVM, underscoring their cross-model stability and suggesting that they represent core metabolic alterations intrinsically linked to the malignant phenotype.

To assess the discriminative potential of these overlapping metabolites, we performed single-metabolite receiver operating characteristic analyses. All six metabolites demonstrated meaningful discriminative capacity with high AUC values, indicating their potential to distinguish tumor tissues from adjacent tissues (Figure 5C). These findings suggest that the identified metabolites may reflect characteristic metabolic alterations associated with endometrial cancer rather than random variation.

## 4. Discussion

Our metabolomic findings indicate that endometrial cancer is shaped not only by genetic or hormonal alterations but also by a systematic reorganization of metabolic priorities. Rather than uniformly activating all metabolic pathways, tumor cells selectively enhance those that provide survival advantages under oxidative stress, inflammation, and rapid proliferation, while attenuating pathways less compatible with malignant evolution [18]. Prior studies have focused largely on serum metabolomics or single metabolic axes, offering only partial insight into tumor-intrinsic metabolism [10,19]. By performing a comprehensive untargeted analysis of paired tumor and adjacent tissues, our study reveals a coordinated redistribution of metabolic resources within the native tumor microenvironment, providing an integrated framework for understanding metabolic reprogramming in endometrial cancer.

Global KEGG enrichment revealed that pathways related to lipid signaling, redox regulation, and nucleotide metabolism were predominantly up-regulated in tumor tissues, whereas nutrient catabolism, aromatic amino acid metabolism, and steroid biosynthesis were down-regulated. This pattern suggests a shift from a physiological homeostatic mode to a tumor-specific stress-adaptive mode, in which cells prioritize metabolic routes that buffer oxidative stress, sustain rapid proliferation, and reinforce oncogenic signaling, while de-emphasizing pathways linked to differentiation or endocrine stability, which consistent with established concepts of metabolic plasticity [11,20,21]. Importantly, these alterations appear coordinated rather than isolated. Teng et al. showed that enhanced lipid signaling can activate pyrimidine metabolism and promote malignant behavior in endometrial cancer, consistent with our observation of concurrent lipid and nucleotide pathway activation [14]. This supports the presence of a lipid–nucleotide metabolic axis in endometrial cancer reprogramming.

Sphingolipid metabolism appears to be an important component of this adaptive strategy. Consistent with previous studies, sphingolipid-related pathways were markedly more active in endometrial cancer than in adjacent normal tissues [22]. Ceramide is a well-known pro-apoptotic lipid capable of triggering mitochondrial permeabilization, necroptosis, autophagy, and ER-stress-mediated cell death [23], and ceramide-modulating agents have been shown to enhance radiotherapy and chemotherapy responses in several cancers [24,25,26]. In our cohort, ceramide levels were significantly elevated in tumor tissues. Rather than signaling impending apoptosis, this increase likely reflects a compensatory response to metabolic and oxidative stress. Tumor cells frequently maintain ceramide at high but sublethal thresholds, enabling it to act as a metabolic and signaling rheostat. Under such conditions, ceramide may support membrane remodeling, modulate stress-responsive signaling pathways, and maintain cellular adaptability, favoring survival rather than cell death.

The enhancement of glutathione metabolism further supports this survival-oriented metabolic shift. Increased ornithine, L-cysteine, and pyroglutamic acid indicate activation of the γ-glutamyl cycle, strengthening antioxidant defenses against persistent ROS stress and creating a microenvironment permissive for continued proliferation and genomic instability [27]. Notably, ornithine elevation has been reported in multiple malignancies, including pancreatic and lung cancers, where it promotes more aggressive phenotypes [28,29]. Inhibition of ornithine metabolism also suppresses endometrial cancer cell growth, suggesting that the increase observed in our samples reflects a recurrent tumor-associated metabolic alteration rather than a context-specific anomaly [30]. Although pyroglutamic acid has not been directly linked to endometrial cancer, it has been associated with protein aggregation and treatment resistance [31], and elevated systemic levels correlate with colorectal cancer risk [32]. Conversely, it is downregulated in gastric cancer, highlighting tumor-type specificity [33]. Its elevation in our cohort likely reflects accelerated γ-glutamyl cycle turnover required to maintain redox homeostasis under metabolic stress.

The systemic up-regulation of arachidonic acid metabolism illustrates a central metabolic crossroads linking inflammation, immunity, and endocrine signaling. The increased levels of PGF2α, 20-H-LTB4, 15(S)-HpETE, and 16(R)-HETE indicate a highly active inflammatory lipid network within the tumor microenvironment. Notably, enhanced arachidonic acid flux has been widely associated with immune suppression and therapeutic resistance across multiple cancers [34,35,36]. These findings suggest that the lipid mediators elevated in our endometrial cancer tissues may contribute not only to inflammatory signaling but also to tumor immune evasion and reduced treatment responsiveness.

The convergence of Random Forest and SVM on six discriminatory metabolites provides new insights into the metabolic network architecture of endometrial cancer. Among them, xanthosine, a nucleoside derived from xanthine and ribose, is well recognized. It has been reported to activate the AMPK/FoxO1/AKT/GSK3β pathway to promote tumor proliferation, and our previous work suggests that xanthosine, as a component of purine metabolism, may contribute to chemoresistance in cancer cells [37,38]. Although the remaining metabolites are either less commonly studied or lack direct evidence linking them to malignancy, their consistent selection by two machine-learning algorithms supports their relevance as robust metabolic features associated with endometrial cancer. For instance, LysoPC (0:0/18:0), an sn-2 stearoyl lysophosphatidylcholine, often reflects enhanced PLA1 or PLA2 activity and increased phospholipid turnover, a feature that may prove mechanistically informative in future studies. Collectively, these metabolites exhibited consistent tissue-discriminative capacity in this exploratory cohort, supporting their value as candidate metabolic features for future validation. Further studies in larger, independent cohorts and evaluation of clinical feasibility will be required to determine their potential utility in endometrial cancer diagnosis.

However, this study has some limitations. First, the relatively small sample size limits statistical power and precludes definitive conclusions regarding diagnostic performance. Although the use of paired tumor and adjacent tissues improves internal consistency, validation in larger, independent, and multi-center cohorts will be required to confirm the reproducibility of the identified metabolic signatures. Second, the machine-learning approaches employed in this study were used for feature selection and prioritization to facilitate discovery of metabolic features associated with endometrial cancer, rather than for constructing or validating clinical diagnostic models. In the absence of cross-validation or independent validation datasets, there remains a potential risk of overfitting, and the results derived from these analyses warrant further confirmation in future studies. In addition, metabolomics as a high-dimensional and highly sensitive analytical approach is inherently subject to methodological challenges, including sample preprocessing variability, batch effects, and complexity in biological interpretation. Some of the observed metabolic alterations may reflect adaptive metabolic remodeling associated with tumor progression rather than direct causal drivers of malignancy, which will require functional studies to elucidate. Finally, the present analysis was conducted at the tissue level, and the functional roles of these metabolic features in endometrial cancer progression have not yet been fully elucidated. Future studies integrating mechanistic experiments and complementary multi-omics approaches will be required to clarify how these metabolic alterations contribute to tumor biology and disease development.

## 5. Conclusions

Our study provides a conceptual framework and experimental foundation for understanding metabolic vulnerabilities in endometrial cancer. By systematically mapping tissue-level metabolic changes and identifying key metabolites consistently prioritized by machine-learning-based analyses, this work highlights metabolic features and pathways that may reflect underlying biological vulnerabilities in endometrial cancer. These findings offer a basis for future mechanistic investigations and translational studies, including validation in larger independent cohorts and evaluation of clinical relevance.

## Data Availability

The original contributions presented in this study are included in the article/Supplementary Material. Further inquiries can be directed to the corresponding authors.

## Supplementary Materials

The following supporting information can be downloaded at https://www.mdpi.com/article/10.3390/biomedicines14010202/s1. Supplementary Table S1: Clinical Characteristics of the Patients.

## Abbreviations

The following abbreviations are used in this manuscript:

QC	Quality Control
PCA	Principal Component Analysis
PLS-DA	Partial Least Squares-Discriminant Analysis
OPLS-DA	Orthogonal Partial Least Squares–Discriminant Analysis
SVM	Support Vector Machine
ROC	Receiver Operating Characteristic
AUC	Area Under the Curve
