# Supplementary material for: Metabolic Landscape of Endometrial Cancer: Insights into Pathway Dysregulation and Metabolic Features
Source: Biomedicines. 2026 Jan 17;14(1):202. doi: 10.3390/biomedicines14010202 (PMC12838860; doi:10.3390/biomedicines14010202)
Supplement: Supplementary file 1 [file biomedicines-14-00202-s001.zip › biomedicines-4071554-supplementary.pdf]

Supplementary Table S1. Clinical Characteristics of the Patients

Patient	Age	BMI	Metabolic disorders	Diabetes	Hypertension	Menopause status	Histological type	FIGO Stage	Neoplasm histologic grade	Neoadjuvant chemotherapy	Radiotherapy	Paired sample	Sample storage temperature
EC2	58	22.0	No	No	No	Pre	Endometrioid adenocarcinoma, NOS	Stage I	G2	No	No	Yes	-80℃
EC3	50	20.0	No	No	No	Pre	Endometrioid adenocarcinoma, NOS	Stage III	G1	No	No	Yes	-80℃
EC4	41	23.6	No	No	No	Pre	Endometrioid adenocarcinoma, NOS	Stage I	G2	No	No	Yes	-80℃
EC5	41	20.3	No	No	No	Pre	Endometrioid adenocarcinoma, NOS	Stage I	G1	No	No	Yes	-80℃
EC6	66	25.2	No	No	No	Post	Endometrioid adenocarcinoma, NOS	Stage II	G2	No	No	Yes	-80℃
EC7	60	23.3	No	No	No	Post	Endometrioid adenocarcinoma, NOS	Stage III	G2	No	No	Yes	-80℃
EC8	65	24.8	No	No	No	Post	Endometrioid adenocarcinoma, NOS	Stage II	G2	No	No	Yes	-80℃
EC10	56	18.9	No	No	No	Peri	Endometrioid	Stage	G3	No	No	Yes	-80℃

							adenocarcinoma, NOS	III					
EC14	48	19.4	No	No	No	Pre	Endometrioid adenocarcinoma, NOS	Stage I	G1	No	No	Yes	-80℃
EC15	57	26.1	No	No	No	Post	Endometrioid adenocarcinoma, NOS	Stage I	G2	No	No	Yes	-80℃

Menopause status: Pre (<6 months since LMP AND no prior bilateral ovariectomy AND not on estrogen replacement); Peri (6-12 months since last menstrual period); Post (prior bilateral ovariectomy OR >12 mo since LMP with no prior hysterectomy).
